# Roles of NRF2 in Fibrotic Diseases: From Mechanisms to Therapeutic Approaches

**DOI:** 10.3389/fphys.2022.889792

**Published:** 2022-06-03

**Authors:** Wenlong Hao, Minghao Li, Qingmin Cai, Shiying Wu, Xiangyao Li, Quanyu He, Yongbin Hu

**Affiliations:** ^1^ Xiangya School of Medicine, Central South University, Changsha, China; ^2^ Department of Pathology, Basic Medical School, Central South University, Changsha, China; ^3^ Department of Pathology, Xiangya Hospital, Central South University, Changsha, China

**Keywords:** Nrf2, antioxidant, fibrosis, KEAP1, oxidative stress

## Abstract

Fibrosis is a persistent inflammatory response that causes scarring and tissue sclerosis by stimulating myofibroblasts to create significant quantities of extracellular matrix protein deposits in the tissue. Oxidative stress has also been linked to the development of fibrosis in several studies. The nuclear erythroid 2-related factor 2 (NRF2) transcription factor controls the expression of several detoxification and antioxidant genes. By binding to antioxidant response elements, NRF2 is activated by oxidative or electrophilic stress and promotes its target genes, resulting in a protective effect on cells. NRF2 is essential for cell survival under oxidative stress conditions. This review describes Kelch-like epichlorohydrin-associated protein 1 (KEAP1)/NRF2 signaling mechanisms and presents recent research advances regarding NRF2 and its involvement in primary fibrotic lesions such as pulmonary fibrosis, hepatic fibrosis, myocardial fibrosis, and renal fibrosis. The related antioxidant substances and drugs are described, along with the mechanisms by which KEAP1/NRF2 regulation positively affects the therapeutic response. Finally, the therapeutic prospects and potential value of NRF2 in fibrosis are summarized. Further studies on NRF2 may provide novel therapeutic approaches for fibrosis.

## 1 Introduction

Fibrosis can be thought of as a maladjusted tissue-healing process brought about by various types of tissue damage. At present, it is believed to be a continuous and almost irreversible dynamic process that occurs most frequently in chronic inflammatory illnesses. The buildup of excessive extracellular matrix (ECM) components, such as fibronectin and collagen, causes the formation of fibrous tissues which, when pathologically disordered, do not lead to successful healing, but rather to progressive fibrosis (Henderson et al., 2020). Pulmonary, liver, kidney, myocardial, and bone marrow fibrosis are the most common types of organ and tissue fibrosis; oxidative stress is one of the critical mechanisms involved (Luangmonkong et al., 2018; Su et al., 2019; Kura et al., 2020; Otoupalova et al., 2020). Excessive reactive oxygen species (ROS) levels promote fibrosis by stimulating organ-specific cell phenotypic changes and increasing extracellular matrix deposition. Various mechanisms are involved in the antioxidant system of the body. The antioxidant response element (ARE) is induced by nuclear erythroid 2-related factor 2 (NRF2), an upstream regulator of the antioxidant response, which drives the transcription of cell-protective genes against oxidative stress and inflammation (Bellezza et al., 2018). Therefore, current studies on NRF2 inducers have attempted to render NRF2 expression to achieve anti-oxidative stress and control the progression of fibrosis.

## 2 KEAP1/NRF2 Signaling Pathway

### 2.1 Main Regulatory Factors

#### 2.1.1 NRF2

NRF2 is a transcription factor that maintains cell homeostasis and generates oxidative damage responses in cells, and is now being investigated as a possible therapeutic target for cancer, inflammation, and fibrotic diseases. By activating or downregulating a variety of proteins, NRF2 can form a hetero-dimer transcription factor (Hozumi Motohashi et al, 2004) with basic leucine zipper proteins (such as small Mafs), which regulate the transcription and translation of more than 500 genes and participate in cellular redox response, inflammation and stress responses, substance metabolism, apoptosis, and autophagy.

**FIGURE 1:**
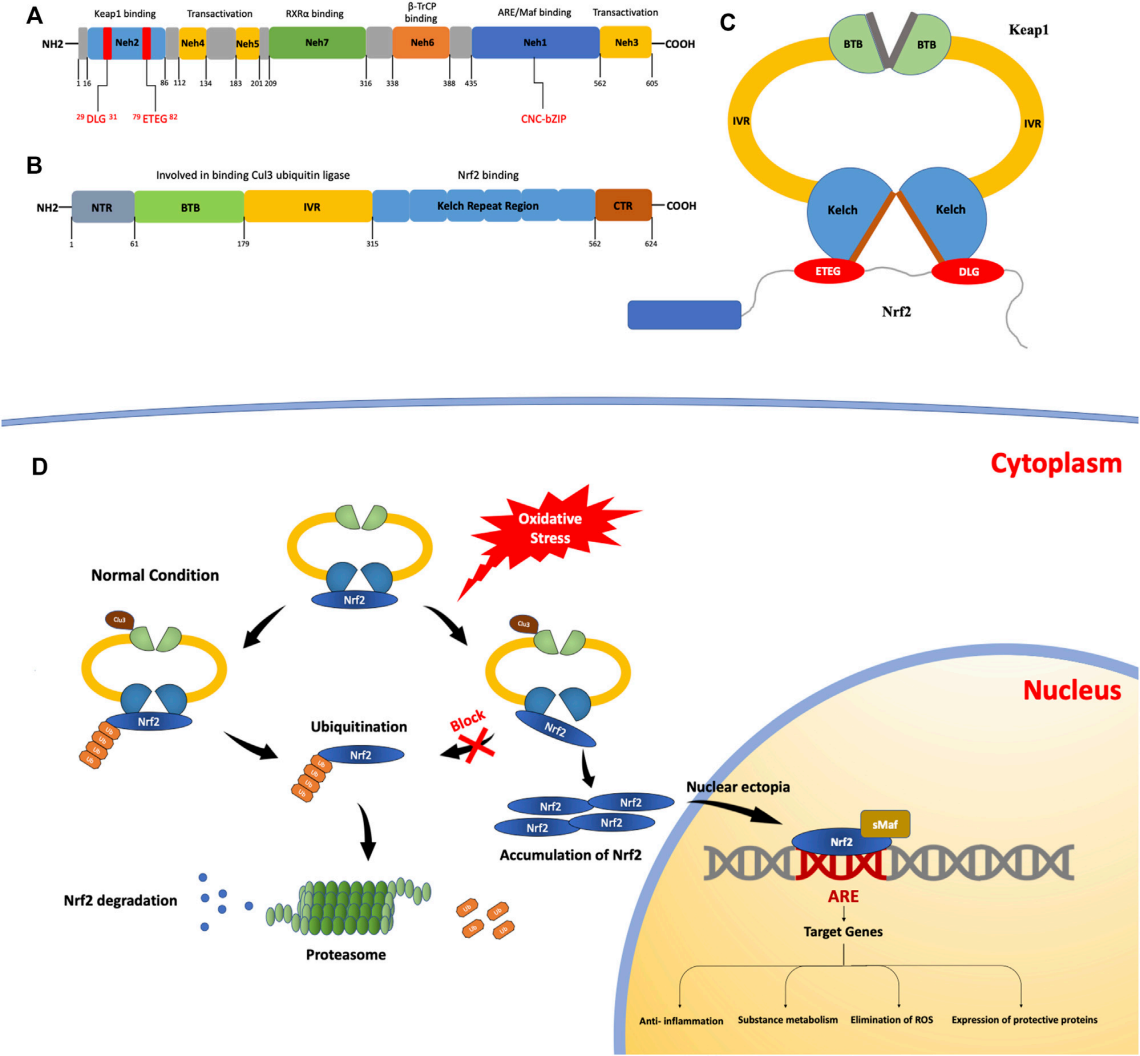
The structures and processes involved in fibrosis. **(A)** Structure of the nuclear erythroid 2-related factor 2 (NRF2) protein. The NRF2-epichlorohydrin (ECH) homology (Neh) 1 domain contains a base sequence of cap-n-collar type basic leucine zipper DNA, which assists in the nuclear transfer of NRF2. The Neh2 domain contains two motifs that bind to Kelch-like ECH-associated protein 1 (KEAP1): ETGE motifs with high affinity and DLG motifs with low affinity. **(B)** The structure of the KEAP1 protein. KEAP1 is a homodimer. **(C)** It contains a Kelch repeat region that combines two motifs (ETGE and DLG) with different affinities to form a hinge–latch structure. The ETGE motif has high affinity and is considered a “hinge”. The DLG motif has a low affinity and can be regarded as a “latch”. **(D)** The KEAP1/NRF2 signaling pathway. Under normal conditions, NRF2 binds to KEAP1 to form an ɑ-helical conformation containing seven lysine residues and then acts as a target for the ubiquitination and cleavage of NRF2. After the ubiquitination of NRF2 by Cullin3 (Cul3), it is degraded and free KEAP1 is involved in the next round of NRF2 binding and ubiquitination. When the oxidation equilibrium is disrupted, the binding of NRF2 and KEAP1 is also affected. The “lock” structure, namely the binding between the DLG motif and the Kelch repeat domain, is destroyed. Therefore, NRF2 ubiquitination is blocked and NRF2 degradation is insufficient. As NRF2 continues to accumulate, free NRF2 is translocated to the nucleus. It can bind with antioxidant response elements to induce the expression of target genes, thus activating the body’s antioxidant defense and anti-inflammatory systems.

NRF2 has a size of approximately 66 kDa (Moi et al, 1994). In this protein, there are seven regional structures with different functions, named NRF2-ECH homology (Neh) domains 1–7, and the C-terminal domains are Neh1, Neh3, and Neh6. The Neh1 domain contains a base sequence of the cap-n-collar type basic leucine zipper DNA, which is essential for NRF2 to bind to Maf family members, bind to DNA as a heterodimer, and enter or leave the nucleus. Studies have found that in yeast two-hybrid screening, the binding of NRF2 and chromodomain helicase DNA binding protein 6 (CHD6) is associated with the Neh3 domain. In addition, the Neh3 domain is involved in activating target gene expression, along with the Neh4 and Neh5 domains. The Neh6 domain is rich in serine and is associated with the Kelch-like epichlorohydrin (ECH)-associated protein 1 (KEAP1)-independent negative regulation of NRF2. During the modulation of NRF2 activity, the Neh7 domain binds to the retinoic acid receptor (a nuclear receptor) and suppresses NRF2 activity (Wang et al., 2013). The Neh2 domain of NRF2 interacts with KEAP1 via two binding motifs: ETGE, with high affinity, and DLG, with low affinity (Figure 1A).

#### 2.1.2 KEAP1

NRF2 is normally produced in the cytoplasm; KEAP1 mediates its ubiquitination and decomposition there and regulates NRF2 through negative feedback (Ulasov et al., 2022). KEAP1 is an E3 ligase-binding protein based on Cullin3 (Cul3), which is approximately 69 kDa (Lu et al., 2016) and contains 27 cysteine residues (Kensler et al., 2007). It can sense the redox state and negatively regulate the activity of NRF2; therefore, it is also known as an NRF2 inhibitor (iNRF2). KEAP1 contains five functional regions: the N-terminal, C-terminal, BTB, IVR, and Kelch repeat domains. KEAP1 binds to Cul3 ubiquitin ligase through the BTB and IVR domains (Pintard et al., 2003). The Kelch repeat region is associated with the binding of KEAP1 to NRF2, which binds to the ETGE and DLG motifs with different affinities and plays a vital role in NRF2 ubiquitination. (Figures 1B,C).

### 2.2 Interaction of KEAP1 With NRF2

At present, there are many models of the binding mechanism of KEAP1 and NRF2; the conformational cycle model (Baird et al., 2014) and hinge and latch dissociation models are popular, and the latter is widely accepted. In this model, NRF2 binds to KEAP1 through the high-affinity ETGE motif and the low-affinity DLG motif; therefore, the model proposes that the former motif can be used as the “hinge” and the latter as the “lock.” (Horie et al., 2021) Using this hinge–lock structure, NRF2 binds to KEAP1 as a homodimer to form an α-helical conformation containing seven lysine residues. Under the action of Cul3, NRF2 is ubiquitinated and degraded (Tao et al., 2017), while the dissociated KEAP1 acts as free KEAP1 and participates in the next round of NRF2 binding and ubiquitination. By binding to KEAP1 and ubiquitination under the action of Cul3 (Lee et al., 2021), NRF2 is continuously produced and degraded in the cytoplasm so that NRF2 can be maintained at a certain level in the cell and isolated in the cytoplasm.

Under oxidative stress, cysteine residues in KEAP1 are covalently modified (Girish et al., 2008), resulting in a conformational change in KEAP1. The conformational changes caused by this modification may have a more significant effect on the low-affinity interaction than the high-affinity one, so the binding of the Kelch domain and the DLG motif, known as the “latch” structure, are destroyed and NRF2 ubiquitination and degradation cannot be carried out normally. Meanwhile, the newly generated NRF2 in the cytoplasm cannot normally bind to KEAP1. Free NRF2 accumulates in the cytoplasm and finally enters the nucleus, where it binds to cis-regulatory elements to induce target gene expression (Hur and Gray, 2011). In summary, when oxidative stress damage occurs, KEAP1-mediated ubiquitination and degradation of NRF2 within the cytoplasm is blocked and cannot be performed. As NRF2 continues to accumulate, free NRF2 is translocated to the nucleus to participate in the transcriptional activation of a series of target genes involved in the activation of defense systems, improvement of cell oxidative stress resistance, and maintenance of the steady state of the cell (Figure 1D).

### 2.3 Function and Mechanism of NRF2

NRF2 mediates the expression of multiple genes and affects various physiological processes, including glutathione synthesis, ROS scavenging, and substance metabolism (Hayes and Dinkova-Kostova, 2014). Increased expression of target genes promotes the transcription and translation of various protective proteins. It enhances the antioxidative stress response of cells, which is of great significance for maintaining cellular homeostasis. Therefore, NRF2 may have preventive and therapeutic effects on various diseases involving the oxidative stress response. In contrast, for malignant conditions such as tumors, NRF2 can be inhibited to weaken the adaptive capacity of cells to oxidative stress and enhance the therapeutic effect of chemotherapy drugs, thus inhibiting disease progression (Sporn and Liby, 2012). In conclusion, NRF2 has great therapeutic significance for diseases (Gorrini et al., 2013), and further research on NRF2 may provide more therapeutic strategies in the clinical setting.

Many studies have shown that there is a KEAP1-independent regulatory mechanism of NRF2 in cells (Bryan et al., 2013; Qin et al., 2014). One study found that when KEAP1 was knocked out in astrocytes, there was still strong expression of the NRF2 target gene when the cells were in a state of oxidative stress; thus, it was speculated that NRF2 might have a substantial effect on astrocytes through a KEAP1-independent signaling pathway. Subsequently, through further experiments, the researchers speculated that the activation mechanism might be related to the direct regulation of NRF2 by the Neh5 transactivation domain (Al-Mubarak et al., 2021). In addition, one study found that in the renal tubules after acute kidney injury, glycogen synthase kinase 3β (*GSK-3β*) overactivity impaired NRF2 antioxidant response via a KEAP1-independent mechanism, resulting in persistent oxidative damages that lead to chronic kidney disease (CKD) (Lu et al., 2019a). The above studies on the regulatory mechanisms independent of KEAP1 have elucidated the regulatory mechanism of NRF2 and provided more comprehensive theoretical support for the achievement of therapeutic goals by regulating NRF2.

## 3 NRF2 and Fibrosis

**FIGURE 2:**
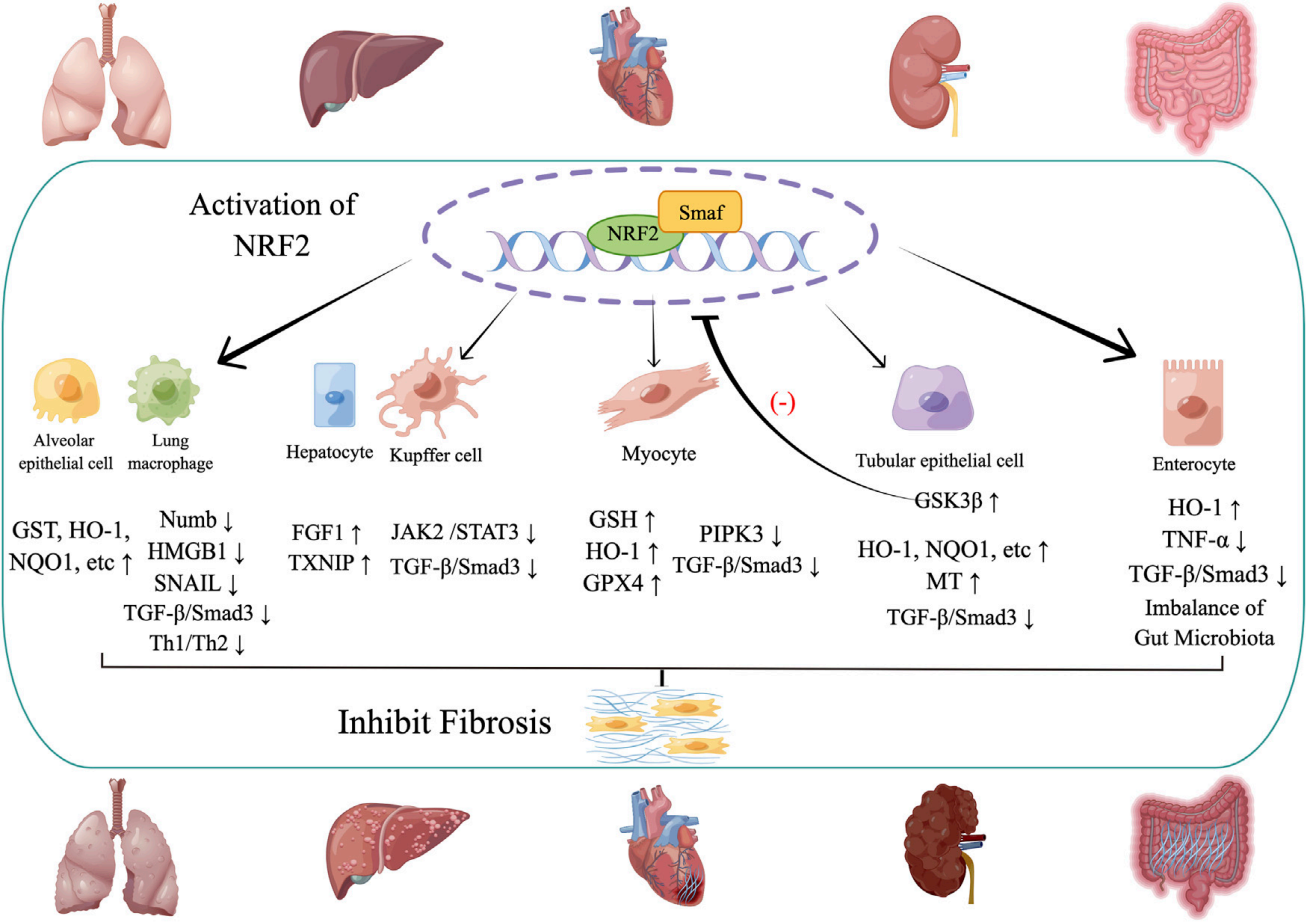
The activation of NRF2 in different organs.

It is well-known that NRF2 plays a significant role in regulating different anti-fibrotic molecules or pathways in other organs. Among various anti-fibrotic factors, the expression of some antioxidant genes (GSTs, HO-1, and NQO1, for instance) are usually upregulated to overcome some oxidant injuries, the most crucial cause of fibrosis. Under the oxidant injuries for a period, fibrotic diseases gradually demonstrate the pathological character of ectopic collagen accumulation. And the natural extracellular matrix may be degraded by abnormal activation of the MMP/TIMP system, which is triggered by a few cytokines existing in the microenvironment of fibrosis, TGF-β, for instance. The two factors above contribute to the genesis of fibrotic diseases. TGF-β/SMADs pathway plays a critical role in the enhanced production and ectopic storage of collagen, which are typical pathological features of various fibrotic diseases. Besides, the abnormal activation of the MMP/TIMP system triggered by TGF-β may degrade the natural ingredients of the extracellular matrix, making space for ectopic collage accumulation. And TGF-β/SMADs pathway can be inhibited by NRF2, resulting in decreased production of collagen and mitigated fibrosis (Henderson et al., 2020). The two anti-fibrotic factors above are most prominent and activated in the most susceptible organs to fibrosis. However, in different organs, other anti-fibrotic mechanisms activated by NRF2 are advanced to assist the two main factors above in relieving fibrosis, downregulation of JAK2/STAT3 in hepatic fibrosis, inhibition of RIPK3 induced mitochondrial dysfunction in myocardial fibrosis, epithelium mesenchymal transition in pulmonary fibrosis and the balance of gut microbiota in intestinal fibrosis for instance (Figure 2).

### 3.1 NRF2 and Pulmonary Fibrosis

Idiopathic pulmonary fibrosis (IPF) is assumed to be caused by a combination of multiple environmental, genetic, and age-related factors, and is an incurable, chronic, and progressive interstitial lung disease with an unclear aetiology (Lederer and Martinez, 2018; Raghu et al., 2018). At present, the root cause of the pathogenesis of IPF is believed to be repetitive local micro-injury of the gene-susceptible alveolar epithelium. Failure of alveolar epithelialization and repair results in aberrant epithelial–fibroblast communication, as well as the recruitment and activation of myofibroblasts (Horowitz and Thannickal, 2006), which produce stroma, large extracellular matrix accumulation, and pulmonary interstitial reconstruction (alveolar tissue bronchiolization) (Yamaguchi et al., 2017; Chu et al., 2020). Oxidative stress is one of the main factors that promote IPF, as it can readily lead to DNA damage, epigenetic changes, abnormal protein balance, mitochondrial failure, and cell senescence, hastening the aging and death of alveolar epithelial cells^1^.

The expression of NRF2, which induces a redox imbalance, protects against IPF. Oxidative damage and acute lung injury are more common in NRF2 deletion mice (Audi et al., 2022). However, the protective effects of NRF2 against IPF require further study. However, it is currently believed that NRF2 works mainly by activating downstream antioxidant gene transcription (GSTs, heme oxygenase-1 [*HO-1*], NAD [P]H quinone dehydrogenase 1 [*NQO1*], glutamate cysteine ligase, uridine diphosphate glucuronosyltransferase, thioredoxin reductase 1, glutathione peroxidase 2, extracellular superoxide dismutase, etc.). Zhang et al. (Zhang et al., 2018b) discovered that avoiding NUMB expression abnormalities by activating the NRF2 antioxidant pathway inhibited epithelial–mesenchymal transformation (EMT) in IPF. Furthermore, NRF2 can inhibit EMT in a variety of ways: for example, transforming growth factor 1 (TGF-1)-induced ROS and EMT generation are attenuated by NRF2 through downregulation of HMGB1 (a novel EMT mediator) (Qu et al., 2019), and NRF2 attenuates EMT and fibrosis by downregulating SNAIL (a significant transcription factor implicated in EMT) expression in IPF (Zhou et al., 2016). By influencing the immune mechanism of IPF, NRF2 can also help alleviate the disease’s advancement. NRF2 can regulate the lung’s balance of Th1/Th2. In NRF2-deficient mice, bleomycin altered the Th1/Th2 balance of the lungs toward Th2 (Kikuchi et al., 2010). Although NRF2 may influence T cell development and cytokine production, it does not affect the homing of Th1/Th2 cells in the lungs. In addition, NRF2 can cause the dedifferentiation of myofibroblasts in IPF. Sulforaphane (SFN) treatment with an NRF2 activator reduced fibroblast oxide in IPF and induced NRF2 expression, antioxidant activity, and myofibroblast dedifferentiation (Artaud-Macari et al., 2013). A recent study discovered that a long non-coding RNA, LOC344887, primarily achieves the anti-fibrosis function of SFN, and the NRF2-mediated upregulation of LOC344887 contributes to the anti-fibrosis potential of SFN, inhibiting CDH2 expression (Liu et al., 2021). However, through immunohistochemical analysis, Mazur et al. (Mazur et al., 2010) identified NRF2 expression in both nuclear compartments of proliferative alveolar epithelial and alveolar epithelial type II cells of IPF patients, but not in the fibroblasts, indicating that NRF2 expression varies between cell types. This is likely to be related to the transformation of fibroblasts into myofibroblasts.

Nonetheless, high-intensity oxidative stress can cause ROS accumulation via the NRF2– Krüppel-like factor 9 (KLF9) pathway, which promotes oxidative stress and exacerbates oxidative damage in cells (Zucker et al., 2014; Gu et al., 2015). KLF9 inhibits the transcription of the thioredoxin reductase 2 (*Txnrd2*) gene, which at least partly leads to ROS build-up in cells. (Chhunchha et al., 2019) reported that NRF2 recruitment is influenced by the dose of H_2_O_2_ or SFN, and NRF2 is recruited to the AREs of *NQO1* and *HO-1* genes at low doses of H₂O₂ or SFN, and to the ARE of *KLF9* at high doses.

In conclusion, although NRF2 has dual effects on IPF, its main effects are anti-oxidative stress and anti-fibrotic.

### 3.2 NRF2 and Hepatic Fibrosis

Hepatic fibrosis (HF) is a degenerative condition caused by the widespread deposition of extracellular matrix (ECM) proteins in injured liver tissue, particularly collagen fibrils (Bataller and Brenner, 2005). Hepatic fibrosis is a typical pathological development in chronic liver injury induced by hepatotoxic and cholestatic injuries, two general chronic liver injuries. It is also an essential step in the progression from chronic liver disease to cirrhosis and hepatocellular carcinoma (Kisseleva and Brenner, 2021).

The mechanisms underlying hepatic fibrosis have been well established. Hepatic stellate cells and Kupffer cells in the liver are activated in distinct ways by injury and inflammation, resulting in biological and functional alterations in the liver tissue (Zhang et al., 2016). Several studies have shown that oxidative stress is a critical process that generates liver cell damage (Tang et al., 2014; Xu et al., 2018). Through its defensive mechanisms, the liver can protect itself from oxidative injury. When the damage caused by ROS created by oxidative stress in the body surpasses the repair capabilities of the internal defense system, cell and tissue damage occur. Activation of the KEAP1/NRF2 signaling pathway has been linked to oxidative stress damage in liver fibrosis in several studies (Galicia-Moreno et al., 2020).

The expression of *NRF2* mRNA is dramatically increased in carbon tetrachloride (CCl_4_) -induced hepatic fibrotic tissues (Yang et al., 2014); conversely, *Nrf2*(L)-KO mice with NRF2 deficiency show more severe CCl_4_-induced hepatic lipid, protein, and DNA damage (Lyu et al., 2020). In addition, by stimulating the TGF-1/Smad3 pathway to activate hepatic stellate cells, stimulate ECM synthesis, and create αsmooth muscle actin (a hallmark of myofibroblast activation), *Nrf2* knockdown has been shown to trigger a cascade of fibrogenic events (Ghanim and Qinna, 2022).

Fatty liver disease is a clinical syndrome characterized by aberrant and excessive fat accumulation in hepatocytes. Non-alcoholic fatty liver disease and alcoholic fatty liver disease are the two types of this syndrome (Benedict and Zhang, 2017; Osna et al., 2017; Loomba et al., 2021). Oxidative stress is one of the key processes leading to hepatocyte steatosis and is essential in the pathophysiology of fatty liver (Ramos-Tovar and Muriel, 2020; Sheka et al., 2020). Steatohepatitis and subsequent fatty liver fibrosis can be caused by a combination of hepatic steatosis and oxidative stress (Loomba et al., 2021). Some studies have revealed that in various obesity and insulin-resistant models, NRF2 activation can reverse insulin resistance, inhibit the steatosis of hepatocytes, and attenuate liver fibrosis (Lee et al., 2014; Yamada et al., 2018; Li et al., 2020). These effects are related to the inhibition of JAK2/STAT3 signaling (Lu et al., 2017), activation of *FGF1* (Lin et al., 2021a), and upregulation of *TXNIP* expression (Yu et al., 2022). It has been shown that the activation of NRF2 using the acetylenic tricyclic bis(cyano enone) TBE-31 can inhibit non-alcoholic steatohepatitis, an advanced form of non-alcoholic fatty liver disease (Sharma et al., 2018). Significant levels of advanced glycation end products are formed in the body during sustained hyperglycemia in individuals with type 2 diabetes, resulting in liver fibrosis (Asadipooya and Uy, 2019; Muthyalaiah et al., 2022). Activation of the KEAP1/NRF2 pathway in the hepatocytes of mice with elevated advanced glycation end products using an adenovirus-associated paradigm dramatically decreases their levels and ameliorates fibrotic damage (Dehnad et al., 2020).

Kohler et al.(Kohler et al., 2014), in contrast, found that activating NRF2 had no significant inhibitory or relieving effects on CCl_4_-induced liver damage and fibrosis; rather, it exacerbated hepatocyte apoptosis and hindered hepatocyte regeneration. This may be linked to the stimulation of *Bcl2l11* and *P15* by NRF2 in hepatocytes.

In conclusion, the KEAP1/NRF2 signaling pathway is a promising therapeutic target for hepatic fibrosis. However, attention should be paid to its inhibitory effect on hepatocyte regeneration when NRF2 activators are applied to treat liver fibrosis.

### 3.3 NRF2 and Myocardial Fibrosis

Myocardial fibrosis destroys the typical heart structure and seriously affects the normal function of heart cells (Li et al., 2018). It is generally believed that NRF2 activation can prevent and alleviate myocardial fibrosis through the alleviation of oxidative stress and inflammatory response (Hu et al., 2018; Kang et al., 2020; Vashi and Patel, 2021). NRF2 may ameliorate fibrosis through the following molecular mechanisms:

After NRF2 activation, glutathione (GSH) production is promoted and myocardial fibrosis is inhibited, limiting the tricarboxylic acid cycle, further inhibiting fibroblast activation, and alleviating myocardial fibrosis. Fibroblast proliferation and activation are critical in the process of myocardial fibrosis, which requires high nutrient levels and energy consumption. To meet the growing demand, glutamate generated by the decomposition of glutamine becomes an alternative carbon source, providing energy for rapidly dividing cells and assisting in the synthesis of nutrients (Altman et al., 2016). Therefore, limiting glutamate production and utilization may be an effective treatment strategy for improving fibrosis (Tsai et al., 2020). A previous study reported that when NRF2 is activated, GSH production is increased. Its production process may competitively consume glutamate, eventually inhibiting fibroblast activation and retarding myocardial fibrosis. Further experiments showed that the effect of the above restriction on myocardial fibrosis was weakened after the deletion of NRF2(Song et al., 2020).

NRF2 activation promotes glucocorticoid receptor (GR) expression and alleviates myocardial fibrosis. A large amount of evidence has shown that the deletion of the anti-aging gene Klotho (KL) is associated with the development of a variety of cardiovascular diseases (Olejnik et al., 2018). In a genetic *Kl*-deficient mouse model, mice showed cardiac hypertrophy, myocardial fibrosis, and other pathological processes, with severe damage to cardiac function (Chen et al., 2021). However, when secreted Klotho protein was added externally, myocardial hypertrophy and interstitial fibrosis were alleviated in *Kl* (−/−) and elderly wild-type mice, and cardiac function was improved. The upregulation of NRF2 and GR was detected in rat ventricular cardiomyocytes cultured with secreted Klotho protein. When GR was overexpressed intracellularly, the researchers found that sclerosing axis A was down-regulated, and the type 1 collagen gene expression was suppressed, leading to a reduction in collagen deposition in fibroblasts. Sclerosing axis A regulates collagen synthesis in fibroblasts and plays a critical regulatory role in fibroblast phenotypes (Bagchi et al., 2016; Zeglinski et al., 2016). However, when the *Nrf2* gene was knocked out by siRNA, the upregulation of GR was eliminated. In conclusion, secreted Klotho alleviated myocardial fibrosis by activating NRF2 to promote GR expression.

One study found that mice exposed to fine particulate matter showed cardiopulmonary damage, which was exacerbated when the *Nrf2* gene was knocked out. A redox imbalance in these mice increased inflammation and stress responses, resulting in myocardial fibrosis and cardiac dysfunction (Chenxu et al., 2020). The loss of NRF2 also promotes the expression of receptor-interacting protein kinase 3 (*Ripk3*) in mice exposed to fine particulate matter, resulting in mitochondrial dysfunction, autophagy, and substance metabolism disorder in mouse cardiomyocytes. However, these changes were not observed in mice with normal NRF2 expression. Considerable evidence suggests that *Ripk3* expression can promote cardiac remodeling and fibrosis, and fibrosis may be improved when *Ripk3* is inhibited (Kazakov et al., 2018). In addition, excessive autophagy of cells can lead to structural and functional disorders of the heart (Dewanjee et al., 2021). Therefore, the researchers hypothesized that NRF2 activation downregulated *Ripk3* and ameliorated mitochondrial disease, thus improving myocardial fibrosis.

In addition, the NRF2 signaling pathway can activate iron death-related proteins (such as GPX4) to inhibit iron death (Dodson et al., 2019; Luo et al., 2021) or reduce ROS production (Tang et al., 2020), inhibit the TGF-β/SMADs pathway, or induce *HO-1* expression (Chen et al., 2019; Li et al., 2019; Yang et al., 2020). Ultimately, it reduces collagen deposition and attenuates thrombin-stimulated CTGF induction, thereby improving cardiac fibrosis.

However, several studies have suggested that NRF2 activation negatively affects cardiovascular events. Some studies have found that when oxidative stress occurs, NRF2-mediated suppression of cardiac autophagy may further aggravate myocardial injury and promote myocardial fibrosis and heart failure. This mechanism requires further study (Zang et al., 2020).

### 3.4 NRF2 and Renal Fibrosis

Prerenal factors play a significant role in CKD and renal fibrosis. Ureteral obstruction and hypoxia caused by renal ischemia lead to oxidative stress and even damage, which may be the mechanism accounting for the diseases above (Su et al., 2019). NRF2 is a well-known factor that plays a prominent role in the hemostasis of oxidation and antioxidation by regulating the expression of the downstream proteins HO-1 and NQO1, for instance (Audousset et al., 2021). It is inferred that abnormal NRF2-related pathways participate in several oxidation injury-induced nephropathies, including CKD and renal fibrosis. In addition, it is known that renal fibrosis may also be triggered by the improper activation of some signaling pathways, such as the TGF-β/Smad pathway, which can also be mitigated by activating NRF2-related gene expression (Meng et al., 2016; Xiao et al., 2019). Kong et al.(Kong et al., 2018) found that NRF2-depleted mice demonstrate increased severity of tubular damage and apoptotic cell numbers after 2 days of ureteral obstruction. More significant tubulointerstitial fibrosis (TIF) promotes the differentiation of fibroblasts to myofibroblasts. It increases fibronectin, and smooth muscle actin occurs 14 days after obstruction, which is always accompanied by decreased antioxidant genes with ARE sequences, which are the downstream genes of *Nrf2*. This supports the theory that NRF2 may play a negative role in the incidence and progression of TIF by activating the expression of downstream antioxidant genes, which may generate antioxidants such as GSH, thus neutralizing the oxidants generated by ureteral obstruction or ischemia and protecting the tubules and interstitium from oxidative damage and secondary TIF. Another study also revealed that oxidative damaged-induced TIF is closely related to impaired NRF2 activity, which contributes to the pathogenesis of oxidative stress (Aminzadeh et al., 2013); this is different from the KEAP1-dependent manner of NRF2 activation in which NRF2 is activated by a structural change in KEAP1, resulting in a protective effect and reduced oxidative damage (Tan et al., 2016).

The KEAP1-independent manner of NRF2 regulation seems to be a cause of antioxidative damage in the kidney. Metallothionein (MT) has been demonstrated to be a potent antioxidant, repairing oxidative damage to vulnerable tissues. Interestingly, MT may also phosphorylate and upregulate the protein kinase B/extracellular signal-regulated kinase (Akt/ERK) pathway in addition to its antioxidant ability, which may activate NRF2, consequently playing a protective role against oxidative damage and induced renal fibrosis (Wu et al., 2015). Furthermore, MT (−/−) mice exhibit more severe hypoxia-induced renal injury and increased accumulation of fibrosis-related factors, fibronectin, and smooth muscle actin, parallel to the inactivation of *Nrf2* downstream genes. Additionally, *GSK3β* is an essential regulator of NRF2 in a KEAP1-independent manner, which plays a role in the negative regulation of NRF2 activation; this has been proven in biopsies, both *in vivo* and *in vitro* (Lu et al., 2019b).

In the exploration of the mechanism by which NRF2 is activated, increasing numbers of natural and synthetic substances are being found to possess the capacity of breaking the “latch” or activating NRF2 in a KEAP1-independent manner, consequently playing a role in protecting against oxidative damage and alleviating renal fibrosis. Therefore, different substances may prevent or mitigate renal fibrosis for various reasons. In *in vivo* and *in vitro* studies, testosterone propionate, dimethyl fumarate, and sinomenine have been found to possess the ability to attenuate renal fibrosis caused by improper activation of the TGF-β/Smad pathway by inhibiting the abnormal expression of TGF-β-induced profibrotic genes or preventing phosphorylated TGF-β stimulation (Oh et al., 2012; Qin et al., 2016; Zhang et al., 2018a). In addition, sinomenine can alleviate oxidative stress by activating NRF2 to upregulate antioxidative genes (Qin et al., 2016). Wang et al.(Wang et al., 2020) found that ureteral obstruction-triggered renal fibrosis can be mitigated by dihydroquercetin, which causes NRF2-related transcription regulation. Therefore, NRF2 is a potential target for developing new therapeutic strategies and the mechanism underlying its role in renal fibrosis should be further explored.

### 3.5 NRF2 and Intestinal Fibrosis

Intestinal fibrosis is a complication of various intestinal diseases, including inflammatory bowel disease (IBD), graft-versus-host disease, drug-induced bowel disease, eosinophilic bowel disease, and radioactive bowel disease, with IBD being the most common cause (Latella, 2018; Lovisa et al., 2019). And it is primarily caused by aberrant ECM deposition resulting from myofibroblast differentiation, recruitment, proliferation, and activation as a result of chronic and recurring intestinal inflammation (Lawrance et al., 2017; Latella, 2018; Lin et al., 2021b). IBD includes Crohn’s disease (CD) and ulcerative colitis (UC). The fibrosis in CD may affect the entire intestinal wall, while in UC the fibrosis affects only the mucosa and submucosa of the large intestine (Lawrance et al., 2017; Lovisa et al., 2019).

Transforming growth factor β1 (TGF-β1) is a well-studied molecule that plays a vital role in the onset and development of intestinal fibrosis. TGF-β1 mainly transmits signals through its downstream SMADs protein family, promoting ECM synthesis and accelerating the fibrosis process (Li et al., 2016; Yun et al., 2019; D'Haens et al., 2022; Hayashi and Nakase, 2022). There is strong evidence that the interaction between NRF2 and TGF-β1 may be one of the key mechanisms contributing to intestinal fibrosis. Guan, Y. *et al.* have revealed that the activation of NRF2 suppresses intestinal fibrosis *in vivo* and *in vitro* through the inhibition of the ROS/TGF-β1/SMAD pathway (Guan et al., 2018a). Similarly, in a mouse model of DSS-induced colitis, a study found that maggot extracts can prevent the progression of digestive fibrosis by increasing NRF2 levels and decreasing TGF-β1 levels (Wang et al., 2021a). Therefore, the activation of NRF2 can inhibit intestinal fibrosis by downregulating TGF-β1 expression. This suggests that NRF2 may be a key target for the treatment of intestinal fibrosis.

Several studies have documented an essential contribution of gut microbiota to the pathogenesis of intestinal fibrosis. Intestinal fibrosis is caused by the immune response and oxidative stress of IBD and may induce dysregulation of the gut microbiota (Liu et al., 2019; Lavelle and Sokol, 2020). It has been demonstrated that UC rats treated with *Lactobacillus* and 5-ASA experience significant improvements in NRF2/HO-1 pathway, reduced the pro-inflammatory factor TNF, and promoted ulcerative colitis recovery, delayed the progression of intestinal fibrosis to some extent (El-Baz et al., 2020). Furthermore, some studies also indicated that NRF2 could inhibit IBD progression to intestinal fibrosis by inhibiting macrophage activation, thereby maintaining the balance of gut microbiota (Kobayashi et al., 2016; Amamou et al., 2022). It is still unclear what role NRF2 plays in gut microbiota in the progression of intestinal fibrosis, but this may suggest new therapeutic strategies.

However, in colorectal cancer, NRF2 has a dual role: it protects cells from carcinogenic damage in the early stages and increases tumor aggressiveness and resistance to chemotherapy in the later stages (O'Cathail et al., 2021; Pompili et al., 2019; Taheri et al., 2020).

In conclusion, NRF2 can not only inhibit intestinal fibrosis but also influence the incidence and progression of tumors (such as colorectal cancer). In this field, further research is required.

## 4 Antioxidant Drugs Related to NRF2

Various studies have been conducted to explore natural or synthetic substances and investigate the efficiency of different NRF2-activating substances, the correspondence between different types of fibrosis and substances, and the methods by which NRF2 is activated. Therefore, many substances have been discovered or are currently being explored. In this section, several effective substances are listed. (Table 1).

**TABLE 1:** Drugs Related to NRF2 in Fibrotic Diseases.

Type of fibrosis	Drugs	Model	Therapeutic mechanism	References	
Pulmonary fibrosis	Pirfenidone (PFD)	Bleomycin-induced lung fibrosis C57BL/6 mice TGF-β1 induced mouse lung fibroblasts	Regulation of NRF2/BACH1 equilibrium, such as the inhibition of BACH1, promotes NRF2 recovery		(Liu et al., 2017)
	Tanshinone IIA (Tan IIA)	Silica-induced lung fibrosis SD rats Silica-induced lung fibrosisWistar rats	Inhibiting EMT and the TGF-β1/Smad signaling pathway and activating NRF2 signaling pathway (silica-induced). By activating NRF2 and inhibiting NOX4, the ROS-mediated PKCδ/Smad3 signaling pathway is blocked to promote the degradation of KEAP1 and convert glutamine into GSH (bleomycin-induced)		(Feng et al., 2020; Zhu et al., 2020)
	Sulforaphane (SFN)	Bleomycin-induced lung fibrosis C57/BL6 mice	By increasing the expression of the NRF2 gene and its downstream antioxidant enzymes		(Yan et al., 2017)
Hepatic fibrosis	Maresin-1 (MaR1)	DEN-induced hepatic fibrosis SD rats	Increasing KEAP1/NRF2 signaling pathway, inhibiting TGF-β1/NF-κB pathway and decreasing inflammatory cytokine expression levels in DEN-induced liver injury mouse model		(Serhan et al., 2000; Dalli et al., 2013; Rodríguez et al., 2021)
	*Pleurotus geesteranus* polysaccharides (PGPs)	Kunming strain mice	Upregulating NRF2/HO-1 signaling pathways in an ALD mouse model		(Song et al., 2022)
Myocardial fibrosis	5,8-Dihydroxy-4′,7 -Dimethoxyflavone (DDF)	Human cardiac fibroblasts	Activating the P38 MAPK/NRF2 signaling pathway, increasing HO-1 expression, and decreasing CTGF expression in HCFs		(Pietta, 2000; Yang et al., 2020)
	Astragaloside IV (AsIV)	ADR-treated SD rats	Activating the NRF2 signaling pathway, inhibiting iron death, significantly improving adriamycin-induced myocardial fibrosis		(Tan et al., 2020; Luo et al., 2021)
	Puerarin	Abdominal aortic banding SD rats	Puerarin downregulates KEAP1, promotes NRF2 expression and nuclear transfer, and can prevent and ameliorate myocardial fibrosis. Its therapeutic effect may be related to the upregulation of UGT1A1 in NRCF by NRF2		(Cai et al., 2018; Zhou et al., 2021)
Renal fibrosis	Salvianolic acid B (SA-B)	Ureteral ligation induced renal fibrosis SD rats	Regulating the differentiation of fibroblasts and modulating the downstream antioxidant genes of NRF2		(Lu et al., 2010; Chen et al., 2014)
	Dimethylfumarate	UUO-induced renal fibrosis C57BL/6 mice	Upregulating the expression of downregulated antioxidative genes (HO-1, NQO1, etc.) and modulating the activation of TGF-β		(Oh et al., 2012)
Intestinal fibrosis	Maggot extract	Human intestinal fibroblasts (CCD-18Co cells) DSS-induced chronic colitis C57BL/6 mice	Up-regulating NRF2 expression at transcription and translation levels, inhibits TGF-β1/SMAD pathway		Wang et al., 2021a
	Tert-butylhydroquinone (tBHQ)	TNBS-induced chronic colitis BALB/c mouse	Changing the conformation of the KEAP1-NRF2 complex, and the DEGRADATION of Nrf2 mediated by Keap1 fails. Increasing nuclear translocation of NRF2		Xu et al., 2017b

### 4.1 Drugs Related to Pulmonary Fibrosis

Pirfenidone (PFD) is currently one of the standard medicines authorized to treat IPF, and its therapeutic effect on IPF is related to the NRF2/BACH1 balance that regulates oxidative stress ability. PFD promotes NRF2 recovery by regulating the NRF2/BACH1 balance, such as inhibiting BACH1 in bleomycin-induced pulmonary fibrosis and TGF-β1-induced rodent models of lung fibroblasts (Liu et al., 2017).

Tanshinone IIA (Tan IIA), which possesses antioxidant, anti-inflammatory, and antifibrotic effects, is the most crucial active ingredient isolated from *Salvia miltiorrhiza*. Tan IIA exerts anti-fibrotic effects by activating the NRF2 signaling pathway to reduce oxidative stress, inhibiting the TGF-β1/Smad signaling pathway, and obstructing EMT. The activation of NRF2 also partially mediates the inhibitory effect of Tan IIA on EMT induced by silica and TGF-β1/Smad pathway activation (Feng et al., 2020). Tan IIA efficiently restored redox homeostasis by upregulating NRF2, inhibiting NADPH oxidase 4, and preventing myofibroblast activation by disrupting the protein kinase C-δ/Smad3 pathway, which is ROS-mediated, decreasing extracellular matrix deposition in rats with bleomycin-induced lung fibrosis. In addition, Tan IIA inhibits KEAP1–NRF2 binding by boosting KEAP1 degradation, resulting in increased NRF2 induction by preserving NRF2 from proteasome destruction and ubiquitination. By activating NRF2, Tan IIA also breaks down glutamine into GSH, reducing glutamate availability in the tricarboxylic acid cycle and preventing myofibroblast activation by inhibiting cell proliferation (Zhu et al., 2020).

The dietary organic sulfur compound SFN, isolated from cruciferous vegetables, is a classic NRF2 activator that exhibits indirect antioxidant action. SFN reduces fibrosis by increasing the expression levels of NRF2 and its subsequent antioxidant enzymes (Yan et al., 2017).

### 4.2 Drugs Related to Hepatic Fibrosis

Maresin-1 (MaR1) is an antifibrotic docosahexaenoic acid derivative (Serhan et al., 2000; Dalli et al., 2013). Numerous studies have shown that it has potent anti-inflammatory properties. Not only can it reduce the production of reactive oxygen species, but inhibit the expression of IL-1β, TNF-α, IL-6, and INF(Saito-Sasaki et al., 2022). Maresin-1 has been shown to increase the nuclear localization of NRF2 regulating the TGF-β1/NF-κB pathway in the livers of mice with diethylnitrosamine-induced liver fibrosis, decrease oxidative stress and inflammation, and stimulate hepatocyte proliferation. (Rodríguez et al., 2021).

*Pleurotus geesteranus* polysaccharides (PGPs), glucopyranoses isolated from *P. geesteranus* mycelium, exhibit liver-protective properties. There is evidence that PGPs may promote cancer prevention, hypolipidemia, antioxidant activity, and hepatoprotection (Zhang et al., 2011). Considering their abundant resources and non-toxicity, they can be potential candidates for the development of food commodities and functional ingredients for new drugs. By upregulating the NRF2/HO-1 signaling pathway, PGPs reduce oxidative stress (Song et al., 2022).

### 4.3 Drugs Related to Myocardial Fibrosis

5,8-Dihydroxy-4′,7-dimethoxyflavone (DDF) is a flavonoid. Flavonoids are antioxidants that are widely present in plants (Pietta, 2000) and have antioxidant effects on various cardiovascular diseases. Based on this, a study associated DDF with myocardial fibrosis and found that DDF can improve fibrosis by promoting nuclear transfer and the phosphorylation of NRF2 to activate NRF2(Yang et al., 2020).

Astragaloside IV is an active ingredient extracted from traditional Chinese medicines. Previous studies have confirmed that astragaloside IV has beneficial effects on many cardiovascular diseases, such as myocardial ischemia and hypoxic injury, myocardial hypertrophy, and myocardial fibrosis (Tan et al., 2020). In rats treated with adriamycin, researchers found that astragaloside IV can downregulate *Keap1*, promote the nuclear translocation of NRF2, reduce collagen deposition, and improve cardiac fibrosis (Luo et al., 2021).

Puerarin is an active ingredient extracted from *Pueraria* root that has been proven to have therapeutic effects on various cardiovascular diseases (Zhou et al., 2021). Studies have demonstrated that puerarin can prevent cardiac fibrosis by downregulating *Keap1* and promoting NRF2 expression and nuclear translocation in mouse models of myocardial fibrosis (Cai et al., 2018).

### 4.4 Drugs Related to Renal Fibrosis

Salvianolic acid B is an organic acid extracted from the Chinese medicine *Salvia miltiorrhiza* (Chen et al., 2014), which has been proven to regulate the differentiation of fibroblasts and modulate the downstream antioxidant genes of NRF2, consequently mitigating the progression of renal fibrosis (Lu et al., 2010).

Dimethyl fumarate, a synthetic substance that may stimulate and activate NRF2, subsequently upregulates the expression of antioxidative genes (*HO-1*, *NQO1*, etc.) and modulates the activation of TGF-β, thus preventing renal fibrosis triggered by oxidative damage and the improper activation of TGF-β(Oh et al., 2012).

### 4.5 Drugs Related to Intestinal Fibrosis

Maggots are a traditional Chinese medicine that has been shown to reduce oxidative stress and reduce inflammatory damage (van der Plas et al., 2009; van der Plas et al., 2007). Researchers observed that weight loss and colon shortening were improved when maggot extract (ME) was applied to mice with chronic colitis. Further studies showed that maggot extract promoted NRF2 expression at the translational and transcriptional levels, thereby improving inflammation-related fibrosis (Wang et al., 2021b).

Tert-butylhydroquinone (tBHQ) is an edible antioxidant widely used in food preparation (Xu et al., 2017a). At the same time, tBHQ is also one of the activators of NRF2. In an experiment to explore the effect of NRF2 on intestinal fibrosis, tBHQ effectively upregulated the expression of NRF2 and improved the anti-fibrosis ability of chronic colitis model mouse cells and human intestinal fibroblasts (Guan et al., 2018b).

While NRF2 has great potential for anti-fibrosis, its activation can also cause some side effects, such as cancer. Some studies have demonstrated that NRF2 plays a dual role in the development of cancer. Lee et al. found that NRF2 can reduce ROS levels and maintain the balance of oxidative stress in normal cells (Lee et al., 2013). Jaramillo and Zhang have revealed that NRF2 can reduce inflammatory cytokines such as TNF-α, IL6, and IL8, reducing inflammatory damage. Overall, NRF2 boosts normal cells’ defenses against carcinogens and prevents cancer from developing (Jaramillo and Zhang, 2013). However, NRF2 has been shown to promote cancer in malignant cells. It may enhance the defense ability of malignant cells and accelerate the development of cancer (Ge et al., 2017; Takahashi et al., 2020). At the same time, many studies have also confirmed that the over-activation of NRF2 is related to the generation of chemical resistance in tumor cells (Furfaro et al., 2016; Li et al., 2021). This effect may be related to the ability of NRF2 to regulate metabolism and antioxidant capacity. Therefore, how to limit the side effects of promoting tumor growth during the application of NRF2 is also an issue that needs to be carefully considered during the study of NRF2. Some scholars believe that it is meaningful to find the transition point of NRF2 from inhibiting cancer to promoting cancer (He et al., 2020), while other scholars have proposed that inhibition of NRF2 downstream protein may attenuate the promoting effect of NRF2 on cancer cells (Cui et al., 2018; Emanuele et al., 2021). However, further research is still needed to ensure the specific mechanisms and effectiveness of these solutions. In summary, we believe that NRF2 is a very potential therapeutic target. Further studies in the future can uncover the multiple mysteries of NRF2 and transform it into one of the powerful therapeutic approaches in clinical practice.

## 5 Conclusion

This review clearly shows that NRF2 plays an essential role in protecting cells from oxidative stress damage. By activating a series of antioxidant genes, NRF2 can inhibit inflammation and oxidative stress responses, improve cell adaptability, and maintain cell homeostasis, which has potential therapeutic significance for various diseases. Therefore, NRF2 activation can be used as a safe and effective therapeutic strategy for fibrosis. However, there is a dark side to NRF2. With NRF2 activation, we will also face some problems such as cancer development. Activated NRF2 not only protects normal cells but also improves the defense ability of malignant cells, which undoubtedly promotes tumor growth. Given the duality of NRF2 expressed in the above studies, further exploration of its possible mechanism of action in different diseases is required to clarify whether NRF2 activation or inhibition is therapeutic in certain situations.
